# Finite element evaluation of dentin stress changes following different endodontic surgical approaches

**DOI:** 10.1007/s10266-023-00882-1

**Published:** 2024-01-06

**Authors:** Tuğrul Aslan, Emir Esim, Yakup Üstün

**Affiliations:** 1 Department of Endodontics, Faculty of Dentistry, Erciyes University, Turhan Baytop Street, No. 1, Yenidoğan District, Talas, Kayseri, 38280 Turkey; 2 Department of Mechatronics Engineering, Faculty of Engineering, Erciyes University, Kayseri, Turkey; 3 Department of Endodontics, Faculty of Dentistry, Erciyes University, Kayseri, Turkey

**Keywords:** Apical resection, Biodentine, Hemisection, Mineral trioxide aggregate, Monolithic zirconia, Root amputation

## Abstract

The aim was to compare the effect of different endodontic surgical treatments on the stress distributions in dentin of a simulated first mandibular molar tooth using the finite element analysis method. Three surgical endodontic procedures (apical resection, root amputation, and hemisection) were simulated in a first mandibular molar. Biodentine or mineral-trioxide-aggregate was used to repair the surgery site in apical resection and root amputation models; the remaining root canal spaces were filled with gutta-percha. Access cavities were restored using resin composite. In hemisection model, root canal was filled with gutta-percha, and coronal restoration was finished with a monolithic zirconia crown. A sound tooth model was created as a control model. An oblique force of 300 N angled at 45° to the occlusal plane was simulated. Maximum von Mises stresses were evaluated in dentin near the surgery regions and the entire tooth. Apical resection/Biodentine and apical resection/mineral-trioxide-aggregate models generated maximum von Mises stresses of 39.001 MPa and 39.106 MPa, respectively. The recorded maximum von Mises stresses in root amputation models were 66.491 MPa for root amputation/Biodentine and 73.063 MPa for root amputation/mineral-trioxide-aggregate models. The highest maximum von Mises stress value among all models was observed in the hemisection model, measuring 138.87 MPa. Hemisection induced the highest von Mises stresses in dentin, followed by root amputation and apical resection. In apical resection, Biodentine and mineral-trioxide-aggregate did not show a significant difference in stress distribution. Biodentine in root amputation may lead to lower stresses compared to mineral-trioxide-aggregate.

## Introduction

The success rate of initial endodontic therapy varies between 53 and 98% when performed for the first time [1–3], whereas the success rate is lower for retreatment cases with a periapical lesion [4, 5]. When traditional root canal treatment or orthograde retreatment choices fails or is not feasible, endodontic surgery keeps the tooth in the mouth. Surgical management includes apical surgery (apical resection), intentional replantation, root resection (root amputation), or crown resection (hemisection, trisection, and bicuspidization) [6, 7].

The aim of apical resection (AR) is to remove the 3 mm of the root structure apically and pathologic periapical tissue; then, a retrograde cavity is prepared, and a biocompatible material is placed into this cavity to seal the root canal system hermetically [8]. AR can be performed to deal with various difficulties of the case as complicated root canal anatomy, presence of persistent periapical infections, a separated instrument that cannot be removed from the root canal, build-ups or posts impossible to retreat, perforations, resorptions, root fractures, etc. [9, 10].

Root amputation (RAM) involves the removal of an individual root from a multi-rooted tooth without removing portions of the crown, whereas hemisection (HEM) results in the complete sectioning of the tooth into separate halves [7, 11]. These surgical treatment options may be considered in the following cases: severe bone loss that affects explicitly one root and cannot be effectively treated using other methods; moderate to advanced furcation involvement with roots diverging in different directions; unfavorable proximity of roots between adjacent teeth; root fracture, perforation, root caries, or external root resorption involving either one root or the furcation area; when performing endodontic treatment on a specific root canal is not feasible and root-end surgery is not recommended; when a tooth, serving as an abutment for a bridge, can be preserved by removing a particular root; or when anatomical factors prevent the placement of a dental implant [10]. Although AR, RAM, and HEM may have different indications and outcomes, their primary goal remains to aid in preserving teeth impacted by endodontic disease.

Mineral trioxide aggregate (MTA) possesses several distinctive properties that make it an excellent material for various endodontic purposes, particularly as a root-end filling during apical surgery. The healing of the surrounding dento-alveolar tissues in response to MTA root-end fillings is remarkable, as it leads to the regeneration of periapical tissues, including the complete formation of apical cementum over MTA [12]. Nevertheless, Biodentine, a promising alternative to MTA as an apical plug material, has been introduced to overcome certain limitations associated with MTA, including complex handling, high cost, long setting time, and potential for discoloration [13]. Biodentine is a calcium silicate-based restorative material that exhibits rapid setting (approximately 10–12 min) and is recommended as a dentin substitute. It can be effectively utilized in various endodontic procedures, such as apexification, internal/external resorption, pulp capping, furcation perforation, and retrograde surgical filling [14, 15].

Finite element analysis (FEA) is vital for evaluating teeth stress distributions and magnitudes. Measuring the mechanical properties of the interaction between biomaterials and biological structures can be difficult. FEA is a numerical method used to analyze the stress and deformation of any geometric structure, yielding results very close to actual measurements [16]. Therefore, it is a powerful technique in the biomechanical analysis of tooth structures when actual conditions cannot be tested in vivo or in laboratory conditions. Endodontic surgical treatments weaken dental tissues; it can be speculated that this may alter the stress distribution in the remaining dental tissues. Stress distributions are often used to predict tooth fracture, because the stress concentration indicates a potential fracture site [17].

Based on the available literature, while some studies have investigated the stress distributions in the remaining root dentin following various surgical endodontic therapies, most of these studies primarily focus on apical resection. No prior FEA study was found specifically investigating hemisection and root amputation in the existing literature. Hence, we assessed the maximum von Mises stress values (a measure utilized to determine whether a particular material will undergo yielding or fracturing) through finite element analysis (FEA) in a simulated tooth subjected to various endodontic surgical techniques and repaired using different methods and materials.

## Materials and methods

### Creation of the FEA models

An extracted intact human mandibular molar tooth with an ordinary root and crown morphology was scanned at a 10-mm voxel size, 125-mA anode current, and 80-kV X-ray tube voltages using a micro-CT device (SkyScan 1272; Bruker, Aartselaar, Belgium). Afterward, the access cavity was prepared on the same tooth with a high-speed handpiece under water cooling. All root canals were enlarged using #25.04 and #35.04, respectively, F360 (Komet, Brasseler GmbH & Co, Lemgo, Germany) nickel-titanium rotary instruments. For the final irrigation, 5 mL of 2.5% sodium hypochlorite, 5 mL of 17% EDTA, and copious irrigation with distilled water were used to make the root canals debris-free. Then, the root canals were dried with paper points. The prepared tooth was subjected to a second micro-CT scanning with the device settings in the previous scan. The bitmap files were configured using NRecon software (version 1.6.3; Bruker, Kontich, Belgium) and then converted into one stereolithography (STL) file with CTAn software (Bruker). STL files were imported to Autodesk Meshmixer software (Autodesk, Inc, San Rafael, CA) to clean the vertex dots inside the tooth, which may produce a problem in surface modeling. The 3-dimensional (3D) surface model with the STL format was converted into a 3D solid model by Geomagic Design X software (Geomagic, Inc, Morrisville, NC). Two canals joining in the middle third of the tooth in the mesial root (Vertucci class 2) and a single canal in the distal root (Vertucci class 1) were observed in the scanned tooth.

### Tested models

#### Apical resection (AR) models (AR/BD and AR/MTA)

**Fig. 1:**
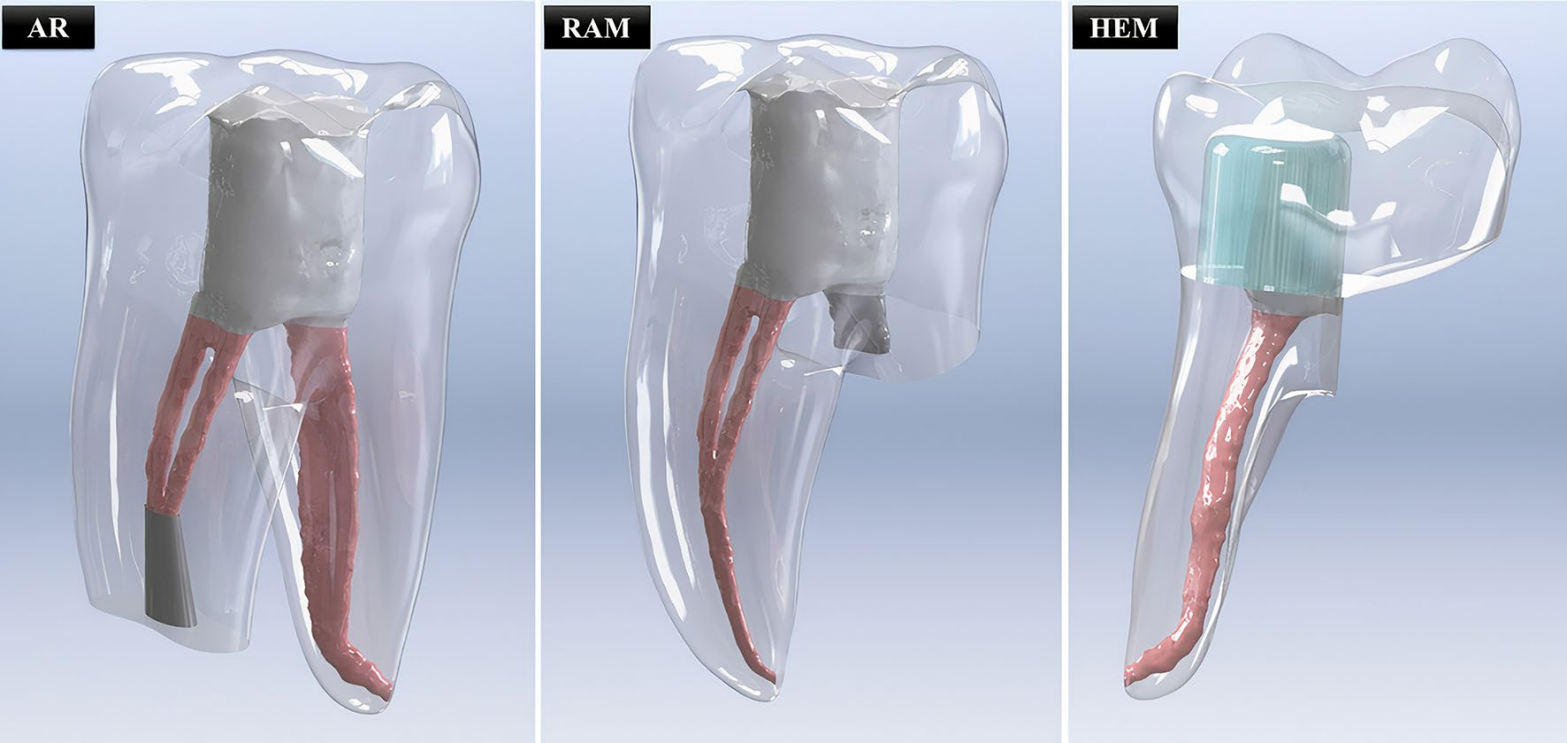
The rendered versions of models created for apical resection (AR), root amputation (RAM), and hemisection (HEM) procedures are shown

3 mm of the root end of the mesial root was removed. A depth of 3 mm of conical retrograde cavity preparation was simulated with a cavity diameter of 1.8 mm at the apical and 1.2 mm at the coronal. The retrograde cavity was filled with BD or MTA. Subsequently, the remaining root canal space of the mesial root and the entire root canal space of the distal canal was filled with gutta-percha, and access cavities were filled with composite resin (Fig. 1).

#### Root amputation (RAM) models (RAM/BD and RAM/MTA)

The distal root was amputated 8.8 mm from the apex, and the remaining root canal (approximately 1.6 mm) was repaired using BD or MTA; the mesial root canal was filled with gutta-percha after the endodontic surgery applied regions were repaired, and access cavities were filled with composite resin (Fig. 1).

#### Hemisection (HEM) model

**Fig. 2:**
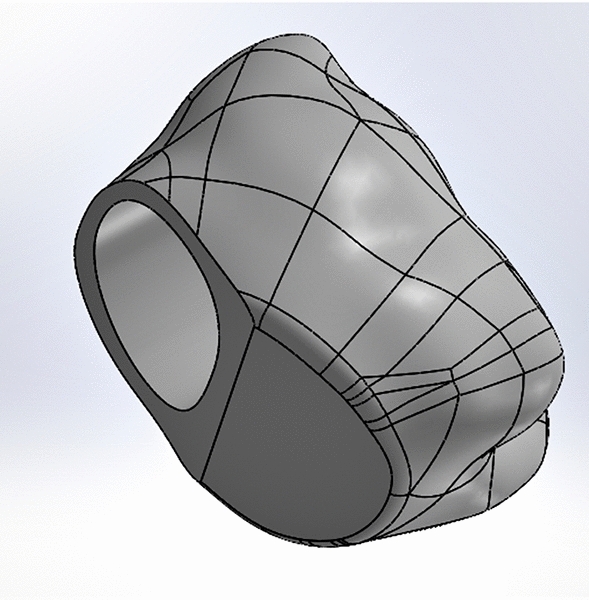
Monolithic zirconia dental crown prepared for coronal restoration of hemisection model

The mesial half of the tooth was removed. The distal root canal was filled with gutta-percha, and the coronal part of the tooth was restored with composite resin. It was prepared as it mimics a prepared tooth crown, and then restored with a monolithic zirconia crown (Figs. 1, 2). The adhesive resin cement used for dental crown was simulated with a thickness of 0.15 mm.

#### Sound tooth (ST) (control)

A sound tooth model without root canal treatment or endodontic surgery was created.

While the ST model was generated based on the 3D solid tooth model acquired from the initial micro-CT scan, the AR, RAM, and HEM models were simulated using the 3D solid model obtained from the second micro-CT scan.

In this study, simulation of surgically treated and fully healed surgical sites was performed in all models. The 0.3 mm periodontal ligament (PDL) thickness around the roots, cortical and trabecular bone, and gingiva was simulated. The root canal sealer was not simulated as it can be neglected due to its very low thickness in canals filled with gutta-percha.

All simulations were performed using SolidWorks software (SolidWorks Corp, Waltham, MA). All components were modeled in contact with each other, so the force applied to the model affects the entire system in the analysis. All simulated models were transferred to ANSYS Workbench, a finite element analysis software (ANSYS, Canonsburg, PA). The “bonded” interface modeling type was preferred between different tissues/materials, because the structures and materials included in the tooth models were structures that form a monoblock and because the materials and structures do not move. The necessary material properties and loading and boundary conditions were defined. All the vital tissues were presumed linearly elastic, homogeneous, and isotropic. The elastic properties of the materials (Young’s modulus and Poisson’s ratio) were determined from the literature and are shown in Table 1 [18–26].

**Table 1:** Material properties used in the finite element models

Material	Young’s modulus (E) (GPa)	Poisson’s ratio (*µ*)
Dentin [18]	18.6	0.31
Enamel [19]	41	0.31
Gutta-percha [20]	0.14	0.45
MTA [21]	11.76	0.31
Biodentine^a^	22	0.33
Resin composite^b^	16.4	0.28
Cortical bone [22]	13.7	0.3
Spongy bone [22]	1.37	0.3
Periodontal ligament [20]	0.0000689	0.45
Pulp [23]	0.003	0.45
Gingiva [24]	0.2	0.45
Monolithic zirconia [25]	205	0.22
Adhesive resin cement [26]	7.3	0.3

^a^Septodont

^b^Kuraray America, Tokyo, Japan

**Fig. 3:**
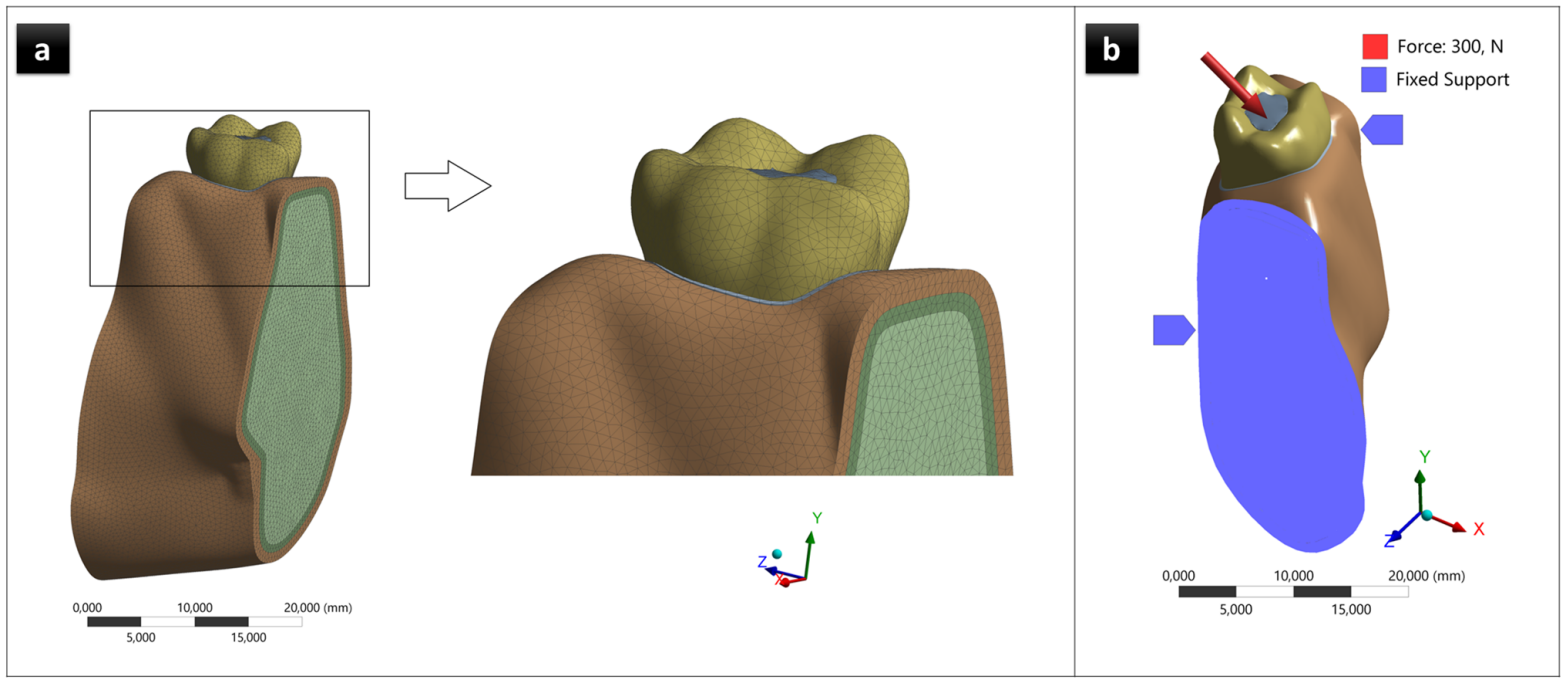
**a** The meshed model. **b** The model of loading conditions. Moreover, the model’s boundary conditions were a fixed support at the mesial and distal surfaces

The convergence test also controlled the accuracy of the FEA models to be validated. In this study, the tetrahedral type of element, with quadratic displacement shape functions and 3 degrees of freedom per node, was preferred in creating the elements. An average of 0.5 mm mesh size was determined to reduce local mesh irregularities and potential artifacts and provide the precision of the resolution in the element dimensions, which are likely to affect the highest stress values (Fig. 3a). After the convergence test, AR models, RAM models, HEM model, and the control model included approximately 807,772 nodes and 539,465 elements, 796,772 nodes and 535,127 elements, 787,510 nodes and 529,031 elements, and 811,227 nodes and 541,088 elements, respectively.

### Loading condition and stress analysis

An oblique force of 300 N angled at 45° to the occlusal plane was simulated and oriented toward the buccal side (Fig. 3b). von Mises stress analyses were performed in root dentin tissue adjacent to the surgery areas and whole tooth dentin tissue. The data were transformed into color graphics using ANSYS software to visualize the stress distributions and magnitudes in the models. The maximum von Mises stresses along the long axis of the root of the models were carefully selected by considering the color scale and recorded.

In addition, the safety factor of all the models under the specified conditions was examined to obtain information about the analyses and to ascertain which treatment methods were the safest. Also, minimum principal stresses (MPS) were evaluated to assess the potential for failure in the material.

## Results

### Maximum von Mises stresses adjacent to the surgery areas of AR, RAM, and HEM models

**Fig. 4:**
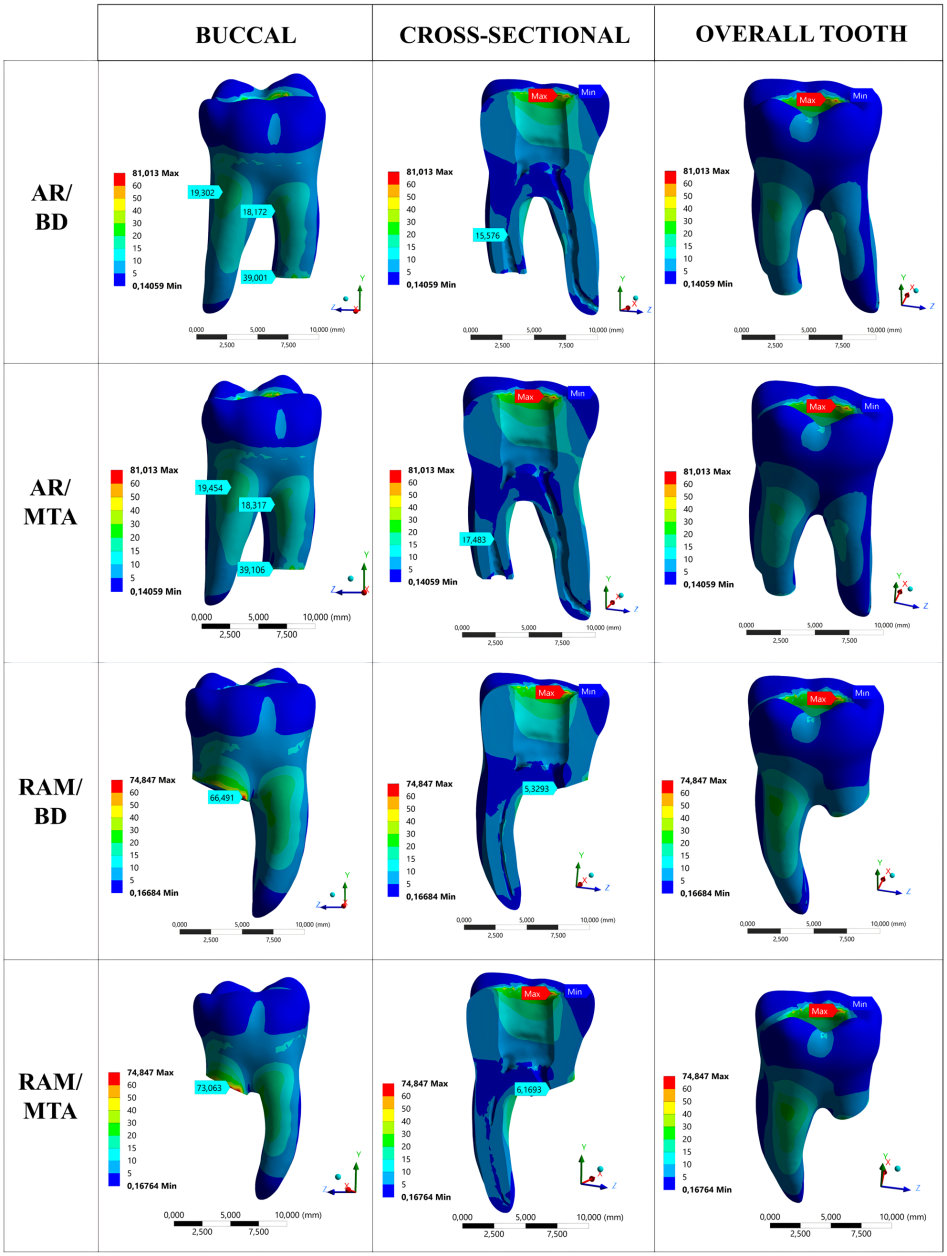
The representation of stress distributions in apical resection (AR) and root amputation (RAM) models from buccal, cross-sectional, and overall aspects

In the AR models, the cut dentin line on the buccal side generated the highest von Mises stresses, with the AR/BD model reaching 39.001 MPa and the AR/MTA model reaching 39.106 MPa. When examining the dentin structure surrounding retrograde cavities, it was observed that there is a slight difference between AR/BD and AR/MTA models, with the AR/BD model exhibiting a lower maximum stress value (15.576 MPa for AR/BD, 17.483 MPa for AR/MTA) (Fig. 4).

The RAM models showed that the cut region of the root had the highest von Mises stresses, specifically in the dentin corresponding to the buccal surface. The maximum stresses recorded were 66.491 MPa for the RAM/BD model and 73.063 MPa for the RAM/MTA model. When examining the dentin tissue surrounding the 1.6 mm space filled with retrograde repair material in the root canal, a slight difference was observed between the RAM/BD and RAM/MTA models, with the RAM/BD model exhibiting a lower maximum stress value (5.329 MPa for RAM/BD, 6.169 MPa for RAM/MTA) (Fig. 4).

**Fig. 5:**
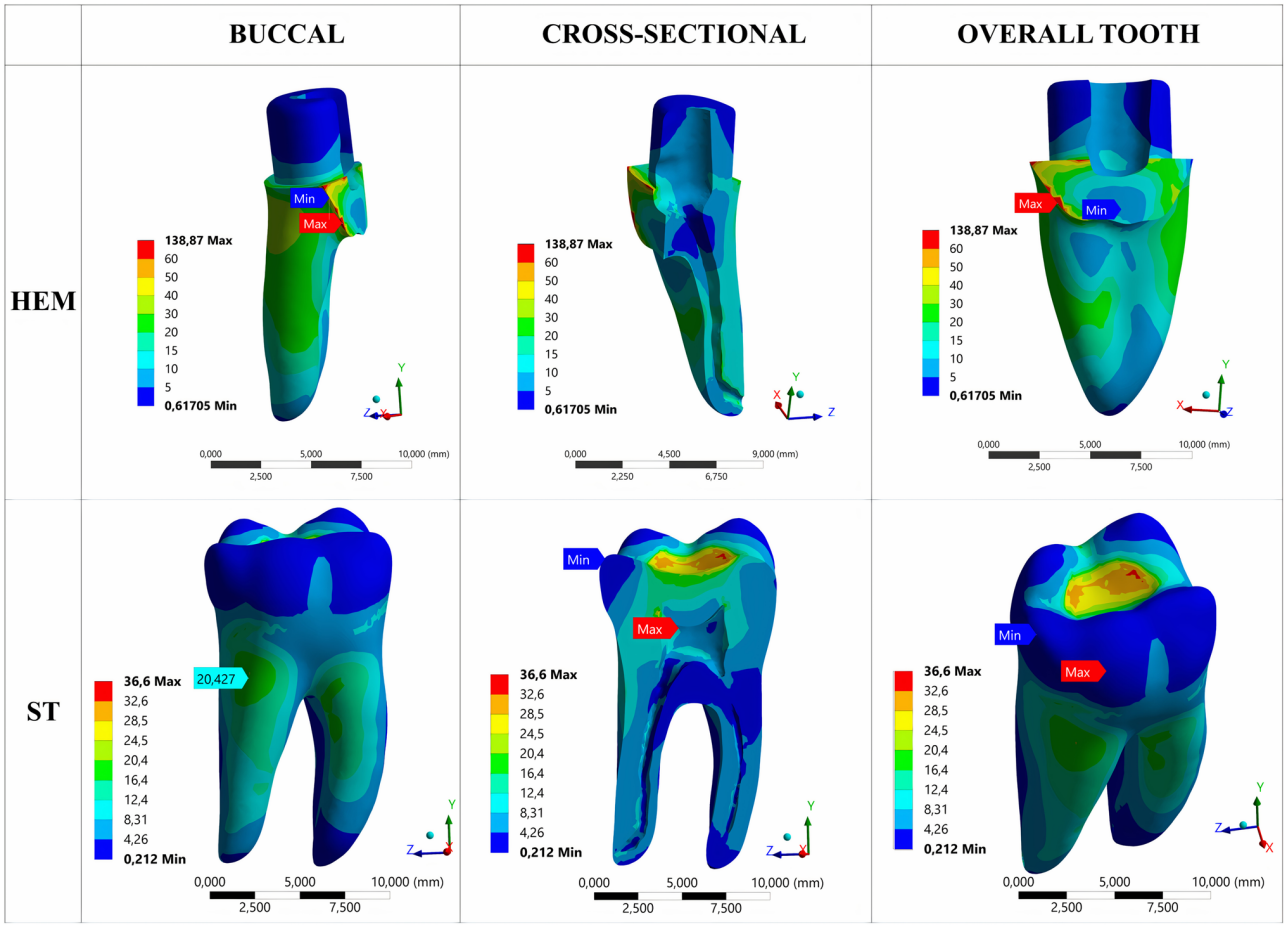
The representation of stress distributions in hemisection (HEM) and sound tooth (ST) models from buccal, cross-sectional, and overall aspects

In the HEM model, the region with the highest von Mises stress was the cervical area, specifically on the buccal surface of the tooth's cut zone, which registered a value of 138.87 MPa (Fig. 5).

### Stress distributions in the aspect of overall tooth dentin structure of the models

The von Mises stress was highest in the cervical region (surgery region) in the HEM model (138.87 MPa) when considering the entire dentin tissue of the tooth. However, the highest stresses were observed in the dentin near the border of the composite filling–dentin junction in the AR (81.013 MPa) and RAM (74.847 MPa) models (Figs. 4, 5).

It was observed that the ST model exhibited the lowest maximum stress value, recorded at 36.6 MPa, with the maximum stresses occurring on the occlusal surface and the pulp chamber roof (Figs. 4, 5).

All maximum von Mises stresses are summarized in Table 2.

**Table 2:** The maximum von Mises stress values, MPS, and safety factors obtained from the finite element analysis models

	Max. von Mises stresses (MPa)			MPS (MPa)	Safety factor
	Cutting line	Dentin tissue surrounding the repair material	Overall		
AR/BD	39.001	15.576	81.013	− 75.96	1.2714
AR/MTA	39.106	17.483	81.013	− 75.96	1.2714
RAM/BD	66.491	5.3293	74.847	− 97.77	1.3761
RAM/MTA	73.063	6.1693	74.847	− 97.778	1.3761
HEM	138.87	–	138.87	− 133.6	0.74171
ST	–	–	36.6	− 48.908	2.81

*AR* apical resection, *RAM* root amputation, *HEM* hemisection, *ST* sound tooth, *BD* biodentine, *MTA* mineral-tiroxide-aggregate, *MPS* minimum principal stress

### Safety factors of the tested models

**Fig. 6:**
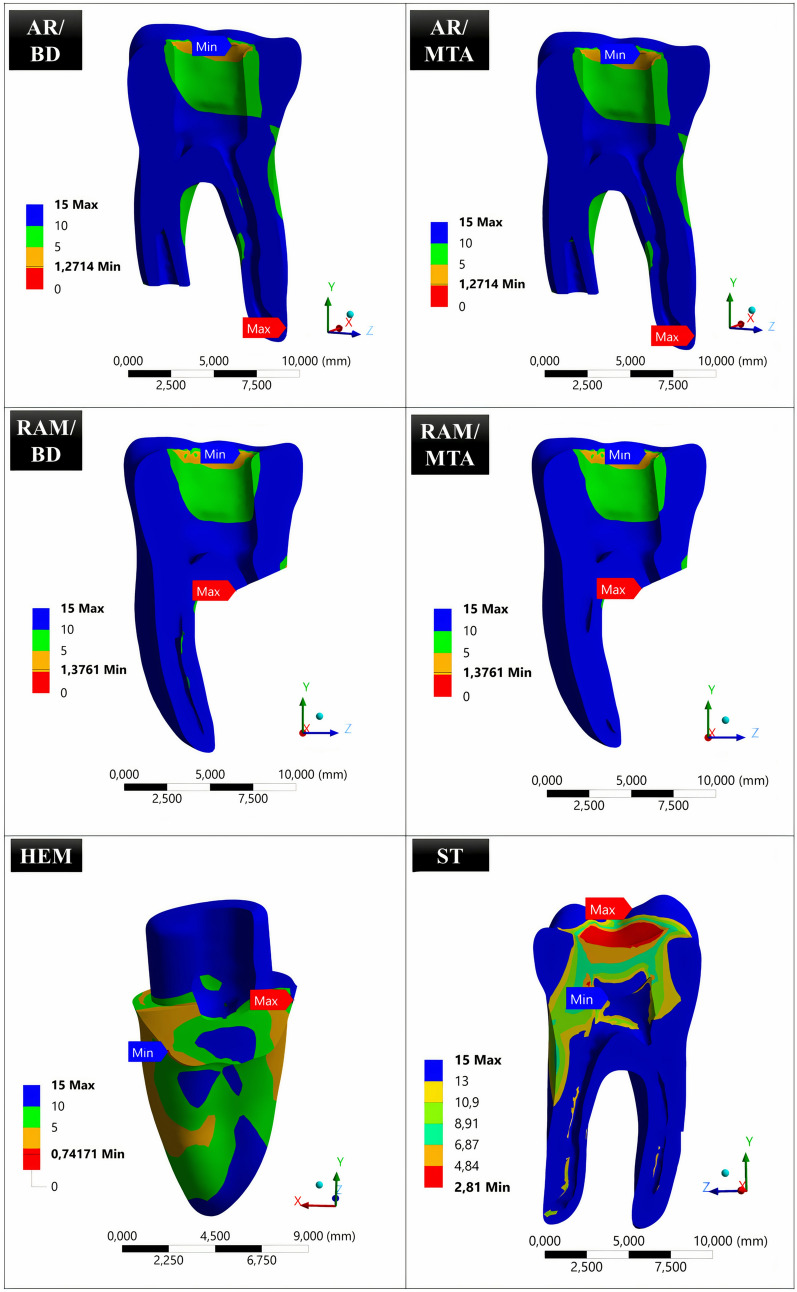
Distribution view of the safety factors in the tested models

The minimum safety factor values in the tested models were 1.2714 for AR/BD, 1.2714 for AR/MTA, 1.3761 for RAM/BD, 1.3761 for RAM/MTA, 0.74171 for HEM, and 2.81 for ST models (Fig. 6, Table 2). Higher values indicate that the system is more secure.

### The minimum principal stresses (MPS) in the tested models

**Fig. 7:**
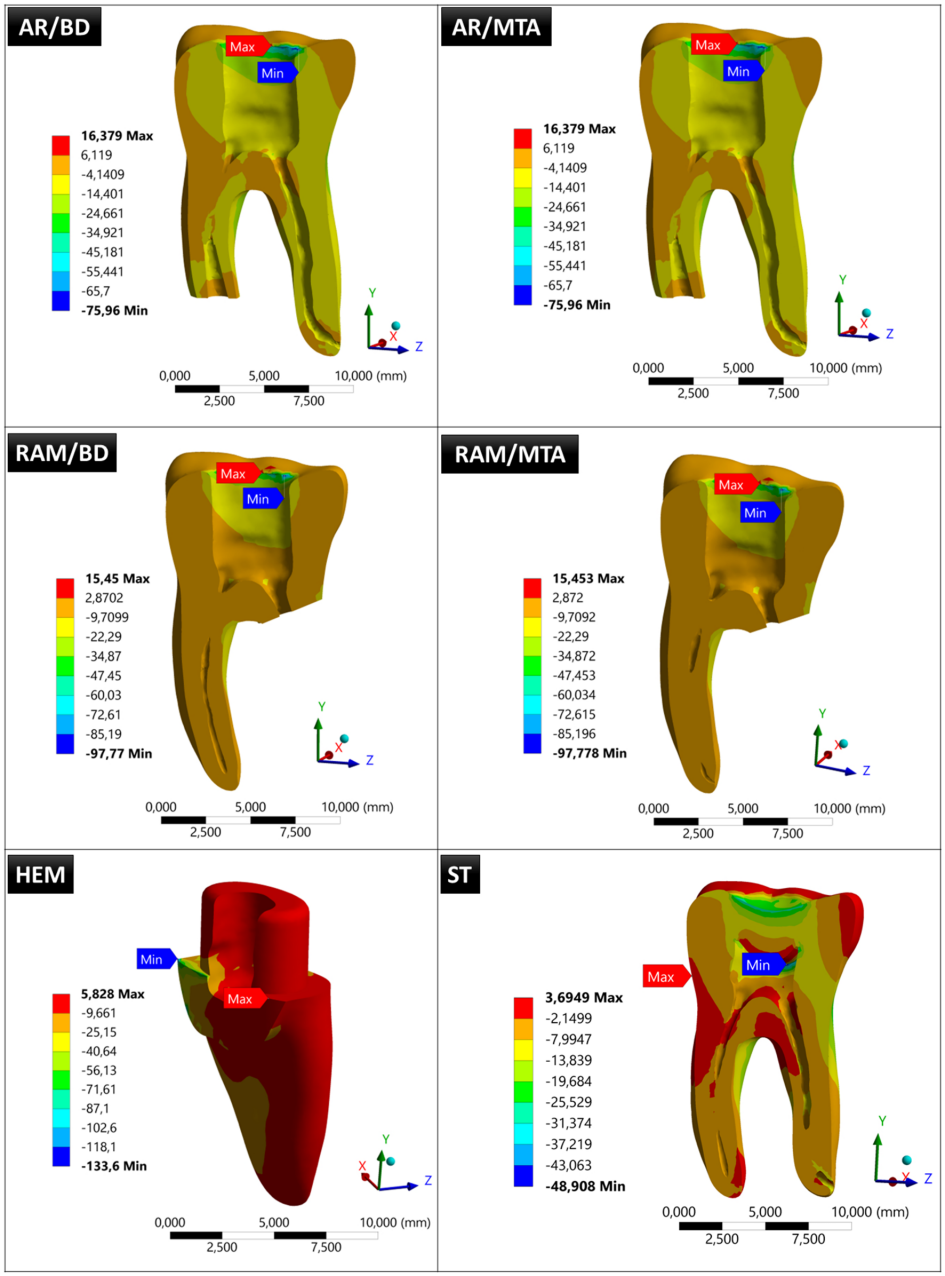
Minimum principal stresses of the tested models

The MPS values in the tested models were − 75.96 MPa for AR/BD, − 75.96 MPa for AR/MTA, − 97.77 MPa for RAM/BD, − 97.778 MPa for RAM/MTA, − 133.6 MPa for HEM, and − 48.908 MPa for ST models (Fig. 7, Table 2).

Absolute values are considered when comparing MPS values.

## Discussion

In this study, we utilized von Mises stresses to represent stress values, since they comprehensively indicate the combined stresses (tensile, compressive, and shear stress components) in the *x*-, *y*-, and *z*-axes [27, 28]. This allowed us to understand the areas of maximum stress in dentin, particularly in the surgery region, which is crucial for identifying potential damage [28]. Identifying areas with the highest stress values in dentin is clinically significant, since it can aid in predicting the occurrence of cracks and fractures that could lead to future complications. Jiang et al. showed that stress concentrations and loading indicate failure initiation sites associated with tooth fracture resistance [29]. Hence, we focused on analyzing the areas with the highest stress values to gain insight into potential failure sites. Additionally, to minimize the impact of local mesh irregularities and avoid artifacts that could affect the highest stress values, we utilized a small mesh size of 0.5 mm after the mesh convergence process.

Hemisection, root amputation, and apical surgery are commonly performed surgical procedures in clinical practice. Although apical resection is a minimally invasive technique involving a small portion of the apex [30], hemisection or root amputation is typically recommended when an entire root is irreversibly damaged. The choice of which half of the tooth to remove, which root to amputate, or which root apex to remove depends on the tooth’s condition and the patient’s needs. Therefore, this study selected one clinical scenario for each surgical type, and models were simulated accordingly.

If the remaining support of the root or roots after the RAM and HEM surgical procedure is not sufficiently strong, splinting to adjacent teeth may arise as an option (splinted crown) [31]. Furthermore, in cases of significant loss of dental tissue in the coronal portion, the application of fiber post and adhesive resin cement may become necessary. Fiber posts are favored due to their elastic modulus, which aligns well with that of dentin, and they exhibit excellent resin adhesion [32]. Thus, achieving a uniform stress distribution along the root structure is possible [32, 33]*.* Adhesive resin cement is frequently used for bonding of fiber posts in root canal treatment, thus resulting in the formation of a monoblock structure. The establishment of a monoblock within the root canal by an adhesive post-core system has been shown to reduce internal stresses within the tooth structure [34]. In our study, we simulated ideally healed surrounding tissues (bone and periodontal ligament) around the remaining roots in all surgical treatments. Moreover, no additional tissue loss was simulated in the RAM and AR models, beyond the access cavity in the crown portion. Therefore, these models were solely restored with a resin composite filling placed in the access cavity. For the HEM model, the coronal cavity was restored with composite resin, and the remaining prepared crown length was simulated to be more than 4 mm (4.2 mm). This aligns with the literature recommendations stating that the preparation length for molar tooth crowns should be at least 4 mm [35]. Furthermore, the prepared tooth crown was simulated to have a composite filling ratio lower than the dentin ratio (approximately 29% for composite resin, 71% for dentin). For these reasons, we designed a single monolithic zirconia crown on the remaining root for the HEM model.

Root canal sealers have been studied for their effect on the fracture resistance of root canals; and majority of studies suggest that root canal sealers can enhance fracture resistance [36–38]. It has been proposed that an optimal root canal filling material should not only bond to the root canal dentin but also reinforce the remaining tooth structure, thereby enhancing the long-term success of endodontically treated teeth [39]. In this context, root canal sealers may play a crucial role, as gutta-percha alone is incapable of forming bonds with the root canal walls [38]. Particularly when resin-based canal sealers are used, a monoblock effect can be achieved, potentially assisting in reducing detrimental stresses in the root [34]. As a limitation, the root canal sealer was not included in the finite element models in our study. The decision to exclude the root canal sealer was influenced by the small scale and thin nature of the sealer thicknesses. The extremely thin root canal sealer layer, combined with geometric complexity, poses challenges in achieving the desired element size and quality, thus impacting evaluation of the results [40]. Therefore, in our study, the thickness of the root canal sealer was neglected.

In the literature, the obturation technique and the instrumentation size have also been assessed regarding the potential effect on fracture resistance of the root canal treated teeth. In a study, Ersoy & Evcil reported that shaping and widening of the root canals reduced the fracture resistance of the teeth, while the use of Thermafill increased the resistance of roots against fracture [41]. Nevertheless, the focus of our study is the examination of stress distribution in teeth following various endodontic surgical procedures. Therefore, in this study, stresses applied by any root canal obturation technique to the root dentin were not simulated. Root canal enlargements were performed up to a size compatible with the preparation size achievable in a typical mandibular first molar, up to #35.04, and no further loss of dentin structure was made.

While hemisection and RAM procedures may appear similar, there is a significant difference in the postoperative crown preparation design. According to the findings of this study, the hemisection model exhibited the highest von Mises stresses among the three different endodontic surgical methods, measuring 138.87 MPa. The significantly higher maximum von Mises stress generated by the hemisection model compared to other surgical technique models may be attributed to the higher elasticity modulus (Young modulus) and hardness of monolithic zirconia compared to composite filling material and dentin tissue (Table 1). This results in minimal absorption of applied forces directly transmitted to the root. Another explanation could be the absence of half of the root and crown of the tooth, resulting in a single root bearing the entire load. Additionally, the unsupported distal half of the monolithic zirconia crown may have caused a moment effect on the mesial root, leading to higher stress increases in dentinal structure.

Following the HEM model, the root amputation method recorded maximum stresses of 66.491 MPa and 73.063 MPa, while the apical resection method yielded values of 39.001 MPa and 39.106 MPa. In a previous study, Wan et al. reported that the stress distributions tend to concentrate in the cervical region of a mandibular molar [42]. They also stated that static loading does not lead to stress concentration at the root apices, which would result in root fracture under normal masticatory loads. The more cervical position of the root-cutting line in the RAM models than in the AR models may have generated higher maximum stresses. Another explanation is that in root amputation, there is a more significant loss of root dentin, resulting in a decreased area to bear the load. As a result, stress values in RAM models may be higher due to the equation “P = F/A” (where P represents pressure, F stands for force, and A denotes area). It is evident that maximum stresses occur on the root-cutting line. This is due to disrupting material continuity and introducing a different material (bone) afterward. As a result, stress accumulations are more pronounced in these areas. Also, the concentration of von Mises stresses on the buccal side of the tooth is primarily attributed to the occlusal force exerted at a 45-degree angle in the buccal direction.

When examining the dentin tissue surrounding the region where the repair material was applied in AR and RAM models, it is evident that the maximum stresses in AR models (15.576 MPa for AR/BD, 17.483 MPa for AR/MTA) are significantly higher compared to those in RAM models (5.329 MPa for RAM/BD, 6.169 MPa for RAM/MTA). Despite the surgical area in RAM being closer to the applied force than AR, higher stress accumulation was observed in the dentin of the root canal wall in AR. This difference mainly arises from the geometric variations between the two models. In apical resection, a retrograde cavity was simulated at the root apex, and maximum stresses were detected at the narrowest region of the cavity where the repair material contacts gutta-percha (transition zone). Therefore, the stresses may be high in this area. However, in root amputation, the canal was left in its original shape at the level where the root was cut, and the repair material had a more comprehensive geometry that did not taper. Consequently, the stresses were lower, since there was more material to support that area when subjected to load. Another reason for this could be the creation of a retrograde cavity in AR models, resulting in thinner dentin walls and higher stresses. According to Ossareh et al., as the amount of dentin removed from the root canal increases, the stress distribution on the root becomes greater [43].

When examining the impact of Biodentine and MTA usage on the maximum stresses in AR and RAM models, it is evident that the maximum stress (66.491 MPa) in the RAM/BD model within the RAM models is lower compared to the RAM/MTA model (73.063 MPa). In addition, when examining the dentin tissue surrounding the areas where the repair material was applied in AR and RAM models, it is observed that the BD models (15.576 MPa for AR/BD, 5.329 MPa for RAM/BD) exhibit slightly lower maximum stress values compared to the MTA models (17.483 MPa for AR/MTA, 6.169 MPa for RAM/MTA). These differences could be attributed to the use of different repair materials. Compared to MTA, as shown in Table 1, the closer elasticity modulus of BD to dentin likely resulted in a more homogeneous distribution of stresses within the surgical area. Moreover, AR models demonstrate almost identical levels of maximum von Mises stress (39.001 MPa for AR/BD and 39.106 MPa for AR/MTA) at the furthest region of the cut root dentin, with Biodentine displaying slightly lower maximum stress. This could be attributed to the factor that the surgical site is distant from the occlusal region where the force is applied. Different repair materials may not have significantly impacted the results at distances far from the occlusal load. In their study, Jang et al. found that apical root resection did not significantly impact the biomechanical parameters of a tooth with a normal periodontium until it reached a length of 6 mm [44].

In this study, the stress distributions within the overall structure of tooth dentin were also evaluated. The maximum von Mises stress concentrations were identified at the interfaces between the composite resin filling and dentin tissue in the apical resection (AR) and root amputation (RAM) models. This situation arises from the proximity of this borderline to the applied force and the presence of a transition zone between the two different materials (dentin and composite). The maximum magnitude of stress recorded in the AR model (81.013 MPa) was slightly higher than that observed in the RAM model (74.847 MPa). This situation may have arisen from the differences in the quantities and types of materials used in the two surgical models. Due to the interactions between these materials under the applied load, such a variation in stress values could have occurred. In addition, the maximum stress value observed in the ST model (control) was the lowest among all models (36.6 MPa). This could be related to the absence of dentin loss in the control model (ST) and, thus, a more homogeneous stress distribution than other models. Santos-Filho et al. reported that removing a significant amount of dentin weakens the root, leading to higher tensile stress levels [45]. When considering overall teeth in the HEM model, it is observed that the maximum stresses coincide with the region where the tooth is cut in half, and it has the highest value among all models (138.87 MPa). Possible reasons for this situation have been mentioned above.

Due to the complex morphology of teeth, the intricate nature of the surrounding root support, and the wide range of bite forces (ranging from 10 to 1000 N) observed among patients, calculating the stresses in a root during masticatory loading is a highly challenging task [42, 46]. To establish an average bite force value, we applied an oblique force of 300 N to our finite element models and analyzed their safety factors [47]. The safety factor can be defined as the tooth-yielding limit ratio to the resulting stress [48], where it indicates the maximum strength of a structure in relation to the applied load. In this study, all tested models, except for the hemisection model, demonstrated a safety factor greater than “1” (Table 2), indicating that these models were deemed safe and secure (at 300 N). However, the hemisection model exhibited a safety factor below “1”. This finding suggests that among the surgical methods tested in this study, the hemisection model is at risk regarding structural integrity (Fig. 6).

Minimum principal stress (MPS) is defined as the maximum magnitude of stress experienced by a material in a specific direction, and it signifies the highest compressive stress level at a particular point within the material. This parameter plays a critical role in assessing the structural integrity, failure potential, and susceptibility of brittle materials to damage, including fracture or fatigue [49]. For brittle materials, researchers often consider the 'normal stress value' in their analyses, because these materials typically fail under normal stresses [49]. Since teeth are also considered brittle materials, it is beneficial to examine normal stresses when evaluating their mechanical behavior. Considering the significant influence of compressive forces on teeth during the chewing process, this study regards normal stresses as the minimum principal stress values. Except for the RAM models, the obtained MPS values in the other models closely align with the maximum von Mises values, indicating the consistency of the results. When assessed from the perspective of the RAM model, an increase in MPS values was observed (Fig. 7). This finding implies a further reduction in the tooth's strength in comparison to von Mises stresses.

However, it is also crucial to perform clinical follow-ups of such surgical treatment options and make interpretations accordingly. Setzer et al. performed a meta-analysis study in which they examined the cumulative outcome rates of crown resections, including hemisection and root resection cases, and they reported that there was no statistically significant difference between the two surgical procedures [7].

## Conclusions

The findings of this finite element analysis (FEA) study demonstrate the following:

1. Hemisection model with a monolithic zirconia crown caused the highest von Mises stresses in the remaining dentin of a mandibular first molar. This procedure was followed by root amputation and apical resection, respectively. Although ideal healing of bone tissue and PDL was simulated in the hemisection model, the destructive stresses observed in the analyses suggest that it may be better to use splinted crowns instead of single crowns. Further stress analysis studies comparing single crowns and splinted crowns after hemisection or even root amputation surgery may contribute to the literature.
2. In both AR and RAM models, maximum stresses were concentrated in the buccal dentin tissue at the cutting line of the root. In these regions, RAM models induced significantly higher maximum stresses than AR models.
3. In root amputation technique, the application of Biodentine may result in lower von Mises stresses compared to the application of mineral-trioxide-aggregate. However, in the case of apical resection, using MTA or Biodentine did not significantly alter the stress distribution. Therefore, the clinical suitability of Biodentine may be more favorable for root amputation procedures.

## Data Availability

The datasets used and analyzed during the current study are available from the corresponding author on reasonable request.
